# The Effects of Psychotherapy on Single and Repeated Ketamine Infusion(s) Therapy for Treatment-Resistant Depression: The Convergence of Molecular and Psychological Treatment

**DOI:** 10.3390/ijms26146673

**Published:** 2025-07-11

**Authors:** Sofia Sakopoulos, McWelling Todman

**Affiliations:** Department of Psychology, The New School for Social Research, New York, NY 10011, USA

**Keywords:** treatment resistant depression (TRD), ketamine infusion therapy, ketamine-assisted psychotherapy (KAP), neuroplasticity, N-methyl-D-aspartate (NMDA) receptor antagonists, rapid-acting antidepressant

## Abstract

Ketamine infusion therapy has gained recognition as an innovative treatment for treatment-resistant depression (TRD), demonstrating rapid and robust antidepressant effects. Its therapeutic promise is increasingly understood to involve molecular and neurobiological processes that promote neural plasticity and cognitive flexibility. These changes may create a unique window for psychotherapeutic interventions to take deeper effect. This retrospective chart review examined the clinical outcomes of individuals with TRD who received either single or repeated ketamine infusion(s), with or without weekly psychotherapy. Depression severity, measured by Beck Depression Inventory scores, was assessed pre-treatment and 30 days post-infusion(s). The results showed significant symptom reduction across all groups, with the most pronounced effects observed in those who received concurrent psychotherapy. While infusion number did not significantly alter outcomes, the integration of ketamine with psychotherapy appeared to enhance treatment response.

## 1. Introduction

Major depressive disorder (MDD) is the leading cause of mental health-related disability and impairment, affecting millions of individuals every year [1,2]. One-third of those affected continue to suffer from depression or become resistant to conventional treatments [3,4]. Current treatment interventions include pharmacological, psychotherapeutic, somatic, and combined methods. Pharmacological options, such as selective serotonin reuptake inhibitors (SSRIs), serotonin–norepinephrine reuptake inhibitors (SNRIs), monoamine oxidase inhibitors (MAOIs), tricyclic antidepressants (TCAs), and atypical antidepressants, frequently exhibit limited efficacy and require long-term courses to establish clinically significant responses. In addition, these approaches are often hindered by other challenges such as negative side effects, dependency concerns, and general ineffectiveness, which contribute to low patient adherence.

MDD has traditionally been conceptualized as a disorder rooted in monoaminergic dysfunction, involving alterations in serotonergic, noradrenergic, and dopaminergic signaling. These neurotransmitter systems are thought to underlie core emotional and cognitive functions such as mood regulation, motivation, and reward processing, and are targeted by first-line antidepressants [5,6]. Despite their widespread use, monoaminergic treatments have significant limitations, including delayed therapeutic onset and a substantial proportion of patients who do not achieve adequate symptom relief, prompting investigation into alternative neurobiological targets [7].

While the glutamatergic system, including the N-methyl-D-aspartate (NMDA) receptor, is not traditionally viewed as a primary etiological factor in MDD, growing evidence implicates glutamatergic dysregulation in the disorder’s pathophysiology. This is especially evident in brain regions central to mood regulation such as the prefrontal cortex and hippocampus [8,9]. Preclinical models have demonstrated that chronic stress can cause NMDA receptor overactivation and dendritic atrophy in these regions, contributing to impaired neuroplasticity [10,11]. Furthermore, neuroimaging studies in patients with MDD reveal alterations in glutamate levels, supporting the involvement of glutamatergic dysfunction in the illness [12].

The clinical efficacy of subanesthetic doses of ketamine, a non-competitive NMDA receptor antagonist, in rapidly alleviating symptoms in refractory presentations of depression provides compelling evidence for the therapeutic relevance of NMDA receptor modulation [13,14]. This has led to growing interest in targeting glutamatergic signaling as a novel treatment strategy for patients who do not respond to conventional monoaminergic treatments, suggesting that NMDA receptor pathways play a key role in the complex neurobiology of MDD.

### 1.1. Treatment Resistant Depression

Despite the fact that 60–70% of individuals with MDD benefit from current treatments, close to 30% of those with MDD do not respond and continue to experience persistent symptoms, leading to an exacerbated presentation of treatment-resistant depression (TRD) [3,15,16,17]. These patients often feel hopeless as conventional treatments fail to alleviate their debilitating symptoms, leading to significant social, occupational, and physical health declines. This highlights the critical need for more effective and targeted treatment options for TRD.

TRD occurs when patients fail to respond to at least two different classes of antidepressants at adequate dosages for six weeks or do not respond to treatments that were previously effective in a succeeding depressive episode [16,18,19]. The underlying biological mechanisms of MDD and TRD remain unclear, with many patients not responding to long-term treatments. Recent studies implicating synaptic plasticity have provided a new avenue for exploring molecular mechanisms and developing effective treatments [20]. Ketamine infusion therapy recently has gained recognition as an innovative treatment for TRD, demonstrating rapid and robust antidepressant effects.

### 1.2. Ketamine

Ketamine has garnered attention as an antidepressant agent due to its rapid and profound antidepressant effects on MDD and TRD. It is an anesthetic substance that produces dissociative and mild hallucinogenic effects, depending on dosage [21,22]. It has been popularized as a psychedelic due to its ability to induce altered states of consciousness, though its mechanism of action differs greatly from traditional psychedelics.

Ketamine was originally developed as an anesthetic in the 1950s, and by 1970, the first FDA-approved ketamine was introduced, marking the beginning of its widespread clinical use, particularly in anesthesia [21]. In 1999, ketamine was classified as a Schedule III non-narcotic substance under the Controlled Substances Act [23]. It is currently FDA-approved for short-term sedation and anesthesia in both humans and animals. Notably, it is indicated for use in pediatric surgery or brief medical procedures that do not require skeletal muscle relaxation because it does not suppress respiration [22,23]. In 2019 the FDA approved esketamine, a nasal spray formulation, for the treatment TRD, marking a significant shift in drug policy regarding the medical use of altered states of consciousness for therapeutic purposes [24]. Currently, ketamine is also widely used off-label for various psychiatric and pain-related conditions [22]. Table 1 shows the therapeutic parameters for ketamine usage including administration routes, dosage ranges, typical treatment protocols, and targeted clinical conditions.

#### 1.2.1. Pharmacology of Ketamine

Ketamine is an arylcycloalkylamine and exists as two isomers, R(−) and S(+) (esketamine), both of which are therapeutically active and makes it a focus of extensive research in psychiatric treatment advancements [21,24]. Ketamine is soluble in water and lipids and can be administered through various routes including intravenous (IV), intramuscular (IM), nasal, oral, and subcutaneous [21]. IV administration, which is considered the ideal route of administration as it is 100% bioavailable, is the focus of this chart review [21]. Recent evidence points to oral and sublingual formulations as potential interventions for wider accessibility in outpatient mental health settings.

#### 1.2.2. Ketamine Mechanism of Action

Ketamine primarily acts as a NMDA receptor antagonist, modulating glutamate through both NMDA and α-amino-3-hydroxy-5-methyl-4-isoxazolepropionic acid (AMPA) receptors [35,36,37,38]. Ketamine blocks NMDA receptors, inhibiting calcium influx, which reduces GABA inhibition and precipitates a glutamate surge [39]. This triggers several downstream effects, including activation of brain-derived neurotrophic factor (BDNF) and the mechanistic target of rapamycin (mTOR), which are involved in neurogenesis and potentiation of synaptic plasticity [20,24,35,37,38,40]. These processes contribute to ketamine’s ability to alleviate depressive symptoms and point to the potential of long-term antidepressant effects.

**Figure 1:**
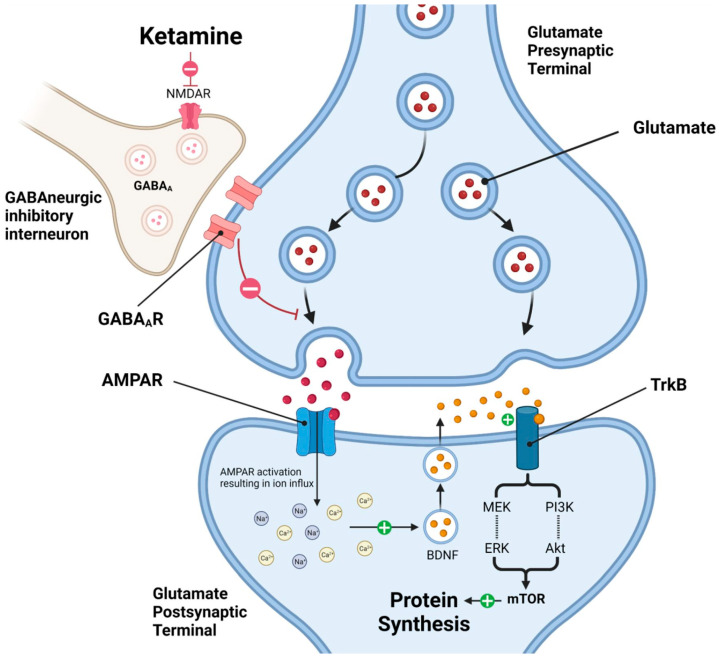
Mechanism of ketamine as an antidepressant (reproduced with permission from [41]).

Additionally, ketamine activates widespread brain regions, likely disrupting dysfunctional brain networks implicated in depression, as shown in neuroimaging studies [40]. These actions suggest ketamine’s potential for enhancing neural function and facilitating recovery in certain psychiatric disorders, particularly depression. Ongoing research into ketamine’s mechanism of action is advancing our understanding of the biological pathways involved in MDD and its antidepressant effects. The mechanism of ketamine as an antidepressant is summarized in Figure 1 (reproduced with permission from [41]).

#### 1.2.3. Molecular Pathways Underlying Ketamine’s Rapid Antidepressant Effects

Building on its NMDA receptor antagonism, ketamine’s downstream molecular effects are likely critical to its rapid antidepressant profile. The disinhibition of GABAergic interneurons initiates a transient surge in glutamate release, which preferentially activates AMPA receptors. This stimulation enhances intracellular calcium signaling, which promotes the synthesis and release of BDNF, a key mediator of synaptic plasticity [42]. Upon binding to its receptor TrkB, BDNF activates the PI3K/Akt and mTOR pathways, driving de novo protein synthesis and structural remodeling at synapses. These neuroplastic changes, particularly evident in the prefrontal cortex and hippocampus, are thought to reverse stress-induced synaptic deficits that underlie depressive pathology [43].

Notably, R-ketamine exhibits more robust and sustained effects than S-ketamine, likely due to enhanced BDNF-TrkB signaling and reduced psychotomimetic properties [44]. In parallel with its synaptogenic actions, ketamine exerts immunomodulatory effects by downregulating pro-inflammatory cytokines such as interleukin-6 (IL-6) and tumor necrosis factor-alpha (TNF-α), mitigating neuroinflammation often associated with depressive states [45]. This anti-inflammatory activity may be especially beneficial for patients with inflammation-associated depressive phenotypes. Furthermore, ketamine may influence epigenetic regulation by modifying DNA methylation and histone acetylation states, altering gene expression patterns in ways that may enhance neural receptivity to subsequent psychological interventions [46]. These multifaceted effects reflect ketamine’s unique role as a rapid-acting antidepressant with both synaptic and systemic implications for emotional regulation. Together, these molecular actions underscore ketamine’s multifaceted therapeutic potential, rapidly engaging synaptic, inflammatory, and genomic pathways to promote neurocircuit restoration and symptomatic relief in depression.

#### 1.2.4. Psychotherapy and Molecular Correlates

Traditionally conceptualized as a cognitive and behavioral process, psychotherapy may have meaningful effects beyond symptom reduction, including influence on biological processes like inflammation and cellular aging. For example, Cognitive Behavioral Therapy (CBT) has been associated with improved regulation of the hypothalamic–pituitary–adrenal (HPA) axis, as evidenced by enhanced cortisol habituation responses [45]. Such changes mirror the neurotrophic and stress-adaptive mechanisms also implicated in pharmacological interventions, underscoring the convergence between psychotherapeutic and neurobiological models of depression recovery.

Furthermore, it seems psychotherapies can also attenuate systemic inflammation. A study found successful outcomes in post-traumatic stress disorder (PTSD) psychotherapy were predicted by functional integrity in fronto-limbic networks, notably decreased amygdala activation and increased dorsolateral prefrontal cortex engagement during implicit emotion processing [47]. These neural predictors suggest that psychotherapy may have the ability to recalibrate maladaptive threat processing circuits, promoting long-term resilience. Further supporting the systematic impact of psychotherapy, psychological stress has also been shown to impact cellular health. In one study, a controlled stress task elicited a measurable increase in telomerase activity, a biomarker of cellular longevity, in immune cells [48]. This transient upregulation, correlated with cortisol levels and perceived stress, suggests that psychological experiences, including those shaped by psychotherapy, can exert direct effects on cellular aging and stress adaptation.

The relevance of these molecular and neural changes is further underscored by longitudinal findings that link psychotherapeutic success to gradual but sustained increases in BDNF levels [49]. While the magnitude and onset of these changes are less immediate than those associated with ketamine, their stabilization over time suggests that psychotherapy may consolidate the neural and molecular adaptations necessary for long-term symptom remission, particularly when pharmacological options are contraindicated or poorly tolerated.

#### 1.2.5. Molecular Synergy in Ketamine-Assisted Psychotherapy (KAP)

Ketamine-assisted psychotherapy (KAP) represents a novel paradigm that synthesizes the rapid neuroplasticity induced by ketamine with the cognitive and emotional restructuring facilitated by psychotherapy. Within hours of ketamine administration, patients enter a neurobiologically malleable state, characterized by increased synaptic formation, decreased inflammatory activity, and epigenetic flexibility, which may enhance the efficacy of psychotherapeutic engagement and deepen therapeutic effects [42,46]. During this window of plasticity, typically lasting 24–72 h post-infusion, psychotherapy may be optimally positioned to influence entrenched cognitive schemas and dysfunctional emotional patterns [50].

Ketamine may transiently reduce the rigidity of maladaptive neural circuits, enabling patients to access difficult psychological material with less resistance. In turn, psychotherapy can leverage this transient plasticity to facilitate reappraisal, emotional integration, and narrative restructuring, processes that contribute to lasting changes in mood and behavior. This integrated treatment approach leverages pharmacological agents to initiate a transient state of heightened neuroplasticity, and psychotherapeutic interventions actively guide the restructuring of cognitive and emotional processes during this window of enhanced neural receptivity [43]. To demonstrate, CBT has been shown to significantly extend the antidepressant effects of IV ketamine in patients with TRD, with responders maintaining remission for up to eight weeks post-infusion [51]. However, CBT offered minimal benefit to non-responders, suggesting that the therapeutic window opened by ketamine must be actively utilized through psychotherapy to achieve lasting clinical gains.

#### 1.2.6. Epigenetic Mechanisms of Ketamine

Emerging research underscores the role of epigenetic mechanisms in mediating ketamine’s rapid and sustained antidepressant effects. Ketamine induces dynamic changes in DNA methylation, histone modifications, and chromatin accessibility within stress-responsive brain regions such as the prefrontal cortex and hippocampus, which are central to mood regulation and affective processing [52]. For example, recent studies have shown locus-specific reductions in DNA methylation at genes involved in synaptic plasticity and immune modulation [52]. Transcriptomic analyses in patients with PTSD and MDD have identified differential gene expression profiles and methylation patterns among responders, with notable regulation of genes such as *PDGFRA*, *CHI3L1*, and *RPH3AL* implicated in neuroplasticity, immune response, and cellular metabolism [53]. Additionally, chromatin remodeling events following ketamine administration appear to increase accessibility at plasticity-related loci, facilitating the transcription of genes critical for synaptic remodeling and emotional relearning [54].

These epigenetic modifications are hypothesized to support both the rapid onset and the durability of antidepressant effects. Longitudinal profiling suggests ketamine may even reverse stress-related biological aging, reflected in reductions in epigenetic aging markers such as GrimAge and PhenoAge [54], indicating a broader rejuvenation of biological systems associated with psychiatric illness. While these findings are promising, important questions remain regarding causality, cell-type specificity, and the long-term behavioral consequences of ketamine-induced epigenetic changes. Addressing these questions will be critical for developing personalized KAP protocols that maximize therapeutic benefit while minimizing risk.

Mechanistically, ketamine’s cascade effects activate downstream PI3K/Akt and mTOR signaling pathways that promote synaptic protein synthesis, structural remodeling, and emotional relearning [52]. These neuroplastic effects are accompanied by histone acetylation and methylation changes that regulate gene transcription involved in synaptic stabilization, affect regulation, and behavioral adaptation. Ketamine also exerts immunomodulatory effects by reducing pro-inflammatory cytokines, potentially mediated by epigenetic mechanisms that suppress neuroinflammatory gene expression [53]. The convergence of these neuroplastic and anti-inflammatory effects creates a transient window of enhanced neural receptivity, offering a critical period during which psychotherapy can consolidate and extend treatment gains.

#### 1.2.7. Non-Ketamine Psychotherapy and Comparative Molecular Profiles

Although ketamine accelerates molecular and symptomatic change, psychotherapy alone can generate comparable shifts over longer periods. Sustained psychotherapeutic interventions have been shown to elevate BDNF levels, normalize HPA axis function, and reduce markers of systemic inflammation, biological changes that parallel those observed with ketamine treatment, albeit on a delayed timeline [45,49]. Moreover, psychotherapies may selectively modulate specific neural circuits associated with emotion regulation and cognitive control, promoting structural and functional reorganization in regions such as the medial prefrontal cortex, anterior cingulate cortex, and hippocampus [47].

In certain clinical contexts, such as patients with contraindications to pharmacotherapy or histories of substance use, psychotherapy may offer a safer and equally effective pathway to recovery. Furthermore, the slower pace of change may confer advantages in terms of integration and sustainability, as therapeutic insights and behavioral adaptations are reinforced over time. Nevertheless, for individuals facing urgent risk or profound treatment resistance, such is the case in TRD, the rapid onset of ketamine’s effects, particularly when integrated with psychotherapy, may provide a critical opportunity for a long-term and integrative intervention.

### 1.3. Ketamine Safety and Tolerability

Ketamine induces an altered state of consciousness that likely facilitates therapeutic change, but it may also cause side effects which points to the critical need to utilize it under medical supervision for maximum therapeutic benefits. Potential side effects may include dizziness, dissociation, nausea, sedation, and changes in blood pressure and heart rate [21,35,36,55]. Repeated use can lead to addiction with psychological dependence and cognitive issues such as memory lapses and executive function impairments [40]. Furthermore, ketamine abuse can lead to lower urinary tract pathologies, cystis, and gastrointestinal symptoms [21,56].

#### Ketamine-Induced Bladder Toxicity

Ketamine-induced bladder toxicity, also known as ketamine-associated uropathy or ketamine-induced cystitis (KIC), is a significant adverse effect primarily linked to chronic, high-dose recreational use but also relevant in clinical contexts. Dose-dependent risk factors include recreational use over 1 g/day, which carries particularly high risk [57], while subanesthetic doses used in clinical settings (typically 0.5–1.0 mg/kg) carry a substantially lower, though not negligible, risk [58].

At the cellular level, ketamine and its metabolites cause urothelial injury, inducing apoptosis and submucosal inflammation. These changes are associated with collagen deposition, smooth muscle atrophy, and fibrosis, reducing bladder capacity and compliance [59,60]. Clinically, this manifests as painful bladder syndrome with urgency, frequency, nocturia, and suprapubic pain [61]. Inflammatory mechanisms involve mast cell activation and elevated pro-inflammatory cytokines such as IL-6 and TNF-α, contributing to chronic mucosal ulceration and edema [60,62]. Disruption of tight junction proteins and depletion of the glycosaminoglycan (GAG) layer compromise the urothelial barrier, increasing permeability and tissue injury [59]. In advanced cases, structural remodeling leads to bladder wall fibrosis, reduced compliance, and elevated intravesical pressures, with risks of vesicoureteral reflux, ureteral strictures, and hydronephrosis [63,64].

Early cessation of ketamine remains the most effective intervention, often resulting in partial or complete symptom resolution, particularly in early stages [57]. Supportive measures include analgesics, anticholinergic or β3-adrenergic agonists, and intravesical GAG replenishment with hyaluronic acid or chondroitin sulfate to restore mucosal defenses [57,63]. In refractory or severe cases with extensive fibrosis or upper tract involvement, surgical options such as augmentation cystoplasty or urinary diversion may be necessary to preserve function and prevent renal damage [63]. Given the multifactorial pathophysiology and chronic course of KIC, multidisciplinary management involving urologists, mental health professionals, and addiction specialists is essential to optimize outcomes. Despite these potential negative side effects, when administered under medical supervision, ketamine can be a highly effective treatment for TRD, offering significant therapeutic benefits.

### 1.4. Ketamine for Treatment Resistant Depression

Ketamine’s effectiveness in TRD is primarily attributed to its unique mechanism of action that distinguishes it from traditional antidepressants. Whereas traditional antidepressants primarily target monoamine systems, ketamine’s mechanism of action centers on the NMDA receptor, a subtype of glutamate receptor [37]. Unlike the monoamine theory of depression, which suggests that a deficiency in neurotransmitters like serotonin and norepinephrine is the primary cause of depression, recent research has implicated the glutamatergic system in the pathophysiology of depression. The glutamate hypothesis of depression posits that an imbalance in glutamate neurotransmission, the primary excitatory neurotransmitter, particularly involving NMDA receptors, contributes to the symptoms of depression [65]. By targeting the NMDA receptor, ketamine provides a novel approach to treating depression that is not addressed by conventional antidepressants. Ketamine’s blockade of NMDA receptors leads to an increase in synaptic glutamate levels, which in turn activates downstream signaling pathways, including the mTOR pathway. This activation promotes synaptic plasticity, neurogenesis, and the formation of new neural connections, which are thought to help reverse the neurobiological changes associated with depression [66].

Furthermore, research suggests that a key mechanism of depression is impaired neuroplasticity, the brain’s ability to adapt and form new connections in response to experiences. Ketamine has been shown to reverse these neuroplasticity impairments, promoting the growth of new synaptic connections and enhancing the brain’s ability to adapt to stress [42,66]. This ability to restore neuroplasticity is particularly crucial for TRD, where traditional therapies often fail to induce the necessary changes in brain structure and function.

#### 1.4.1. Dissociation

One of ketamine’s key features is production of dissociative effects, which may also play a vital role in its antidepressant properties. Studies suggest that ketamine-induced dissociation, such as derealization and depersonalization, might contribute to its therapeutic outcomes, particularly in certain subtypes of depression [67,68,69,70]. These effects are likely due to an enhancement of glutamate release, with individuals experiencing greater dissociation potentially exhibiting increased presynaptic glutamate release in response to the ketamine dose [71].

The dissociative experiences are thought to disrupt maladaptive thought patterns contributing to depression. By inducing a temporary disconnection from typical patterns of thought and emotion, ketamine may facilitate psychological plasticity, allowing patients to break free from rigid and negative thinking [67,70,72]. This effect may be particularly helpful for individuals with TRD who have become entrenched in maladaptive behavioral and cognitive patterns that are resistant to change through traditional therapeutic approaches. However, the relationship between dissociation and antidepressant response is complex and not fully understood, with some studies reporting no significant association [69,72,73].

#### 1.4.2. Rapid Onset

One of the most significant advantages of ketamine over traditional antidepressants is the rapid onset of antidepressant action. While conventional antidepressants, such SSRIs, often take weeks to show clinical effects, ketamine has been shown to produce rapid antidepressant effects, often within hours of administration [74]. This fast-acting nature is particularly beneficial for patients with TRD, who are resistant to treatment options and may suffer from severe symptoms, including suicidal ideation, requiring immediate relief and higher need for care [75,76]. The rapid antidepressant effects are likely due to its ability to enhance glutamate release, resulting in a surge of glutamate in the synapses [40].

#### 1.4.3. Antidepressant Effects of Ketamine on TRD

Numerous studies have shown that a single low-dose (subanesthetic) ketamine infusion (0.5 mg/kg) can rapidly and significantly improve depressive symptoms, including suicidality in TRD [75,76,77,78,79,80]. The rapid antidepressant effects of subanesthetic doses of ketamine have been consistently confirmed in both animal models and in individuals with TRD, with effects peaking around 24 h and lasting up to seven days [20,75,77,79,80,81,82,83]. Despite ketamine’s rapid antidepressant effects, the potential transient nature of these effects has led researchers to explore repeated infusion protocols in an effort to sustain antidepressant benefits.

Repeated ketamine infusions, typically administered as six infusions over 2–3 weeks, demonstrate rapid and significant antidepressant effects in MDD and TRD, even after fourteen days post-infusion [75,79,80,81,82,84,85]. Some research indicates that repeated ketamine infusions may maintain and prolong therapeutic outcomes [74,85]. Further evidence shows that repeated ketamine infusions produce rapid and enduring antidepressant effects in individuals with TRD, with effects lasting over 30 days post-infusions [86,87]. While psychotherapy combined with ketamine shows mixed results, some research suggests it may enhance long-term effects by facilitating emotional insights and reframing pathological beliefs with neuroplasticity [75,79,88]. Despite these promising findings, several questions remain regarding the long-term efficacy, optimal dosing schedules, and safety of repeated ketamine infusions to inform effective and safe treatment protocols.

This study addresses a gap in the literature by examining the antidepressant effects of single and repeated high-dose (1 mg/kg) ketamine infusions at 30 days post-infusion(s), with and without weekly psychotherapy. It was hypothesized that both single and repeated high-dose (1 mg/kg) ketamine infusions would produce sustained reductions in depressive symptoms at 30 days post-infusion(s), and that the addition of weekly psychotherapy would prolong these antidepressant effects compared to ketamine infusions alone.

## 2. Results

### 2.1. Clinical Characteristics

In the ketamine infusion and psychotherapy groups, ages ranged from 19 to 66 years old (*M* = 41.4, *SD* = 14.3) and in the ketamine infusion and no psychotherapy groups, ages ranged from 21 to 65 years old (*M* = 43.3, *SD* = 15.1). In the psychotherapy groups, the majority were female (64%) and recreationally drug-naive (76%); similarly, in the no psychotherapy groups, the majority were female (70%) and were recreationally drug-naive (80%).

Baseline BDI-II scores indicated varying levels of pre-treatment depression severity across groups. In the single infusion groups, most patients presented with moderate depression (46%), followed by mild (33%) and severe depression (20%). In contrast, the majority of patients in the repeated infusion groups exhibited severe depression at baseline (78%), with the remaining classified as moderate (22%). None of the participants had a documented history of substance abuse or substance use disorder. In both the single infusion with psychotherapy group and the repeated infusion with psychotherapy group, two individuals were diagnosed with Bipolar II Disorder. Additionally, two individuals in the repeated infusion with psychotherapy group had depression with psychotic features.

### 2.2. The Effects of Psychotherapy on Single and Repeated Ketamine Infusion(s) Therapy for TRD

A comparison of pre- and post-infusion BDI-II scores for the entire sample revealed a significant reduction in BDI-II scores (*Mpre* = 28.83, *Mpost* = 20.58), *F*(23) = 125.03, *p* < 0.001. A separate 2 × 2 Analysis of Covariance (ANCOVA) ([psychotherapy vs. no psychotherapy] × [single infusion vs. repeated infusions]) with the post-induction BDI-II scores as the DV and pre-infusion scores as the covariate revealed a significant main effect for Psychotherapy (*Mnopsy* = 23.18, *Mpsy* = 18.99), *F*(19) = 10.83, *p* < 0.004, but not for the number of infusions.

### 2.3. Depression Categorizations Pre- and Post-Infusion(s)

In the single infusion group, most patients presented with mild (33%) or moderate (46%) depression at baseline, as indicated by their BDI-II scores. Following the single ketamine infusion treatment, with or without psychotherapy, the majority of participants shifted to categories of clinically “normal” (46%) or mild depression (33%). In the repeated infusion groups, the majority of patients initially fell within the severe depression range (78%). After completing the treatment protocol, the distribution shifted, with 11% categorized as having mild depression and 33% having moderate depression, with 44% remaining in the severe range. Notably, while four patients (44%) in the repeated infusion groups continued to fall within the severe category, all showed reductions in their BDI-II scores from baseline.

Table 2 presents changes in BDI-II depression severity classifications from pre- to post-infusion(s) for each patient across the single and repeated infusion groups, with and without psychotherapy. Despite initial differences in baseline depression severity between the groups, every participant experienced improvement and showed a reduction in BDI-II scores 30 days post-treatment. These results support ketamine infusion therapy as a potent antidepressant intervention for individuals with TRD.

### 2.4. Tolerability and Safety

No serious adverse events requiring discontinuation or safety concerns were noted in the chart review. One participant reported mild nausea, which was successfully managed with ondansetron post-infusion.

## 3. Discussion

Given the significant burden of TRD and the limitations of conventional clinical interventions, identifying an effective and enduring treatment option remains a critical need. Ketamine has emerged as a promising candidate, offering rapid and robust antidepressant effects in patients unresponsive to traditional pharmacotherapies. Over the past decade, subanesthetic low-dose (0.5 mg/kg) ketamine infusions have consistently demonstrated rapid symptom reductions, often within hours, effects that standard antidepressants typically fail to achieve over several weeks [75,76,77]. These outcomes have been replicated across animal models and human studies, underscoring ketamine’s transformative role in depression treatment [20,81].

While a single infusion may alleviate symptoms for up to a week [80], repeated infusions over 2–3 weeks have been associated with more sustained effects and reductions in suicidal ideation, indicating a treatment intervention for more severe presentations [75,76,78]. However, relapse following treatment cessation remains a concern, prompting the need for optimized maintenance strategies and adjunctive interventions [38,81]. A growing area of investigation involves the use of higher ketamine doses. While most studies rely on the standard 0.5 mg/kg dose, emerging evidence suggests that high-dose infusions (1 mg/kg) may lead to more durable antidepressant outcomes without corresponding increases in adverse effects [77,87]. For example, Sakopoulos and Hinz (2024) [87] found that single and repeated high-dose infusions in conjunction with weekly psychotherapy led to significant symptom reductions at 30 days post-treatment. The underlying mechanisms likely involve increased glutamate release and NMDA receptor antagonism, which in turn enhance synaptogenesis and neuroplasticity [75].

In addition to its neurobiological effects, ketamine’s dissociative properties may enhance introspection and emotional processing, potentially contributing to its antidepressant efficacy [71]. These altered states may offer a unique opportunity for patients to engage in psychotherapy, particularly when rigid cognitive and emotional patterns inhibit progress in standard therapeutic contexts.

Combining psychotherapy with ketamine infusion therapy has emerged as a promising approach for TRD. This integrative method, often referred to as KAP, leverages the rapid antidepressant effects of ketamine alongside the enduring benefits of psychotherapy [89]. Ketamine is believed to moderate maladaptive belief patterns associated with depression. When combined with psychotherapy and its promotion of neuroplasticity, it may enhance the therapeutic process and contribute to sustained antidepressant effects [50,69]. Ketamine can facilitate a therapeutic state conducive to psychotherapy, shifting pathological psychological systems, enhancing neuroplasticity, and promoting introspection. In a study by Dore et al. (2019) [50], the authors reported significant improvements in depression and anxiety scores among participants receiving sublingual and IM KAP, suggesting that the combination may be more effective than ketamine or psychotherapy alone. Moreover, the timing and structure of psychotherapy relative to ketamine administration appear critical, some advocate for therapeutic engagement during the acute psychoactive phase [90], while others emphasize the importance of preparatory and integrative sessions to consolidate insights and promote lasting change [87,91].

Ketamine’s antidepressant effects are primarily mediated through NMDA receptor antagonism, which triggers a cascade of downstream events, including AMPA receptor activation, increased glutamate release, and upregulation of BDNF and mTOR signaling, that promote synaptogenesis and neural plasticity, particularly in prefrontal and limbic regions involved in mood regulation [51]. At a psychological level, ketamine’s dissociative effects may temporarily disrupt entrenched patterns of self-criticism, allowing for greater emotional openness and perspective shifts, conditions that psychotherapy can harness to foster lasting change.

The present retrospective chart review sought to evaluate the 30-day post-treatment effects of single and repeated high-dose (1 mg/kg) ketamine infusion(s), with and without concurrent weekly psychotherapy. All patients who received psychotherapy did so with the same practitioner. Psychotherapy sessions were conducted within 24–72 h post-infusion using a psychodynamic-relational approach to integrate ketamine experiences into a meaningful framework for change. Sessions focused on exploring emergent material, examining maladaptive thoughts and behaviors, deepening narrative coherence, and helping patients recognize new possibilities for emotional connection, self-understanding, and behavioral shifts to address TRD. Consistent with existing literature, the findings demonstrate that both single and repeated infusion(s) significantly reduced depressive symptoms in individuals with TRD. Importantly, both ketamine and psychotherapy independently contributed to symptom improvement, with psychotherapy exhibiting a significant main effect in ANCOVA analyses. Participants across all four treatment conditions showed significant reductions in BDI-II scores at 30 days post-treatment.

The majority of patients in the repeated infusion groups began with severe depression, yet many experienced a shift to moderate or mild depression post-treatment, even in the absence of psychotherapy. Interestingly, although repeated infusions did not yield statistically superior results compared to a single infusion in this sample, improvements were observed in both groups. This raises important questions regarding individualized dosing schedules, especially in the context of baseline symptom severity and personal factors such as treatment access or cost-related constraints. Patients with more severe symptom presentations tended to self-select into the repeated infusion protocol, and their higher baseline BDI-II scores suggest that the severity of their depression may have influenced this treatment choice. Despite this, significant improvements in both single and repeated groups lend further support to the enduring antidepressant efficacy of high-dose ketamine infusion treatment.

Crucially, psychotherapy appeared to enhance outcomes, with a significant main effect of psychotherapy on BDI-II score reduction found, supporting the hypothesis that ketamine’s neuroplasticity window can be leveraged through therapeutic engagement. All psychotherapy was delivered by the same clinician, minimizing therapist-related variability and strengthening the inference of psychotherapy’s additive benefit.

To date, few studies have systematically examined the effects of high-dose ketamine with and without psychotherapy at a 30-day follow-up. The current study contributes to an emerging body of work, suggesting that high-dose ketamine, particularly when paired with weekly psychotherapy, can be a powerful and enduring intervention for TRD. As conventional antidepressants fail as an effective intervention for a large proportion of patients with TRD, ketamine-based interventions, especially at higher doses and in combination with psychotherapy, represent a novel and promising step toward more rapid, individualized, and biologically informed care. These findings underscore the clinical potential of ketamine as part of a multimodal strategy, bridging molecular-level brain change with psychotherapeutic engagement to address complex depressive presentations.

### 3.1. Limitations

This study has several notable limitations. First, its retrospective and observational design limits the ability to establish causal inferences about the therapeutic effects of ketamine infusion therapy. A randomized, double-blind, placebo-controlled trial would have been the gold standard to rigorously evaluate efficacy while controlling for expectancy effects, placebo response, and other confounding variables. Additionally, the study utilized a small convenience sample drawn from a private outpatient clinic, which included only individuals who could afford to pay out-of-pocket for treatment. This financial barrier may have introduced selection bias and limited the generalizability of the findings to more diverse or economically diverse populations. Although the study’s design reflects clinical practice and offers valuable preliminary insights into novel treatments for TRD, the small sample size (*N* = 24) limits the robustness of the conclusions and generalizability. Furthermore, while depressive symptoms were assessed at 30 days post-infusion(s), the durability of ketamine’s antidepressant effects beyond this time point remains largely unknown.

Moreover, the broad age range of participants (19–66 years) represents an important consideration in interpreting these findings. NMDA receptor function and glutamatergic neurotransmission undergo age-related changes, including declines in receptor density and alterations in subunit composition [92,93]. These variations can affect both the pharmacodynamic response to NMDA receptor antagonism and the tolerability of ketamine treatment. Although adolescents were not included in the current sample, differences between younger and older adults in NMDA receptor characteristics may contribute to heterogeneity in therapeutic outcomes. Finally, the use of a single clinician to deliver all psychotherapy sessions represents a limitation. While this approach reduces variability from differences in therapist style, technique, and interpersonal dynamics, it also introduces a potential confounding therapist effect. This limits the generalizability of the findings, as replication with other clinicians may yield different results. Therapist effects are well-established in psychotherapy research and can account for a substantial proportion of outcome variance [94].

### 3.2. Directions for Future Research

To further establish ketamine as a viable and sustainable treatment for TRD, future research should expand across multiple domains such as molecular, clinical, psychotherapeutic, and systemic. While current findings highlight ketamine’s rapid and robust antidepressant effects, the neurobiological and therapeutic mechanisms underlying these changes remain incompletely understood. From a molecular perspective, the precise pathways responsible for ketamine’s antidepressant efficacy merit deeper exploration. Though the modulation of glutamatergic signaling is widely recognized as central to ketamine’s action, downstream effects involving synaptic plasticity, neurotrophic factor regulation (e.g., BDNF), and anti-inflammatory processes remain active areas of investigation. Research that elucidates these molecular cascades, particularly how they interact with acute subjective experiences such as dissociation, could inform the development of next-generation therapeutics that replicate ketamine’s efficacy with fewer side effects or logistical barriers.

One major area of research interest should be optimizing the enduring antidepressant effects of ketamine infusion therapy in order to provide a safe and effective intervention for TRD. Despite promising developments, several critical questions remain including the ideal frequency, duration, or dose of ketamine infusions to maintain long-term remission. More randomized controlled trials are needed to compare low- versus high-dose regimens, single versus repeated protocols, and the timing of psychotherapeutic interventions. Furthermore, the long-term safety of high-dose, repeated ketamine administration remains unclear. Potential side effects, including cognitive impairments, bladder toxicity, and increased risk of substance misuse, underscore the need for rigorous longitudinal assessment [83].

Equally important is the development of standardized clinical guidelines for KAP. Currently, variability in psychotherapy modalities, session timing, and integration practices makes it difficult to generalize findings or establish best practices. Research into the neurobiological correlates of therapeutic change during KAP may offer crucial insights for tailoring interventions to individual patients.

Most current clinical protocols employ low-dose ketamine (typically 0.5 mg/kg IV), but emerging interest in a broader dose spectrum, including psycholytic (sub-psychedelic) doses, warrants further investigation. Psycholytic doses may induce subtle alterations in consciousness conducive to emotional processing without the full dissociative effects associated with higher doses. Future studies should examine the efficacy, safety, and experiential quality across different doses to determine how dose-dependent factors relate to long-term outcomes, therapeutic alliance, and integration with various psychotherapy modalities (e.g., psychodynamic therapy, cognitive-behavioral therapy, or experiential/arts-based interventions). Particular attention should be given to therapeutic timing (e.g., preparatory vs. integrative psychotherapy), modality, and frequency, especially in maintenance protocols combining repeated infusions with ongoing therapy. A valuable line of inquiry would also assess depressive symptoms at extended follow-up intervals (e.g., 60, 90, and 120 days post-infusion), to better characterize the durability of ketamine’s effects and identify optimal maintenance schedules.

Finally, broader efforts should aim to increase access to KAP by addressing systemic barriers such as cost, clinic availability, and provider training. Inclusion of more diverse patient populations, including those with comorbid psychiatric or medical conditions, will be critical in evaluating the generalizability and safety of ketamine therapy across real-world contexts. Future research may benefit from adopting a transdisciplinary approach; bridging neurobiology, psychopharmacology, and psychotherapy to deepen understanding of ketamine’s antidepressant mechanisms and translate this knowledge into personalized, accessible, and enduring treatments for individuals with TRD.

## 4. Materials and Methods

### 4.1. Participants

Patient charts from a private psychiatric practice were reviewed for individuals with TRD who received the relevant treatment protocols over a two-year period (*N* = 24): single ketamine infusion with weekly psychotherapy (*n* = 9), repeated ketamine infusion with weekly psychotherapy (*n* = 5), single ketamine infusion without psychotherapy (*n* = 6), and repeated ketamine infusion without psychotherapy (*n* = 4). All patients receiving weekly psychotherapy did so with the same practitioner before, during, and 30 days after the infusions.

### 4.2. Measures

#### 4.2.1. Beck Depression Inventory

The Beck Depression Inventory, second edition (BDI-II) is a 21-item self-report inventory that measures depression symptom severity in adults and adolescents [95]. Each item is rated on a 4-point scale from 0 to 3, with the summation of score ranging from 0 to 63 indicating the severity of depression. Summed scores fall into depression categories ranging from mild (14 to 19 points) to severe (29 points or more) depression. The BDI-II is a reliable and valid screening tool for depression widely used in clinical practice and research [96].

#### 4.2.2. Antidepressant Treatment History Form

TRD was defined as the failure to respond to at least two different pharmacological classes of antidepressant medications, at adequate dosages for at least 6 weeks during a depressive episode, or failure to respond to previously effective treatments in a succeeding depressive episode [16,18,19]. TRD was verified in the patient chart using the Antidepressant Treatment History Form (ATHF) [97]. This is a formalized method of evaluating treatment adequacy and resistance that involves checking off each pharmacological class of antidepressant, brand of drug, length of treatment; along with other treatment modalities like psychotherapy over course of depression treatment.

#### 4.2.3. Inclusion Criteria

Patient charts were reviewed to ensure all met the DSM-5 criteria for MDD, single or recurrent episode, as deemed by the physician who completed a structured clinical interview [98]. Participants met DSM-5 criteria for either unipolar (*n* = 22) or bipolar depression (*n* = 2), with depressive episodes serving as the primary treatment focus. As part of the standard intake process at the private psychiatry practice, all participants were screened for a history of substance use disorders; none had a documented history of drug or alcohol abuse. In addition, baseline BDI-II scores of ≥14 were required for inclusion, indicating the presence of at least mild depressive symptoms. TRD was confirmed using the Antidepressant Treatment History Form (ATHF), which documented trials of at least two antidepressants from different pharmacological classes, including SSRIs, SNRIs, TCAs, MAOIs, and atypical antidepressants.

#### 4.2.4. Exclusion Criteria

Patients who had received ketamine infusion therapy within the past 30 days were excluded to reduce potential confounding effects.

### 4.3. Study Design

This retrospective chart review analyzed BDI-II scores pre and 30 days post ketamine infusion(s) in patients who met TRD criteria and completed one of the four treatment protocols: single or repeated (6) ketamine infusion(s) with or without weekly psychotherapy. Patients eligible for ketamine infusion therapy were prescribed repeated infusions with weekly psychotherapy, though some chose the single infusion protocol or no psychotherapy due to factors other than clinical, such as cost and time. Weekly psychotherapy sessions were scheduled within 24–72 h post-infusion(s) to coincide with the window of heightened neuroplasticity. Sessions focused on processing infusion experiences and supporting therapeutic goals. During treatment, patients received subanesthetic high-dose (1 mg/kg) intravenous ketamine, with the repeated infusions occurring over 2–3 weeks [77]. BDI-II scores were collected two days before and 30 days after the infusions for analysis. Patients remained on prescribed pharmacological treatments during ketamine infusion therapy, including the 30-day follow-up period. These included SSRIs (*n* = 8), SNRIs (*n* = 3), atypical antidepressants (*n* = 2), and mood stabilizer/antipsychotic (*n* = 7). The remaining patients (*n* = 4) were not on any other pharmacological support other than the ketamine treatment.

Data were analyzed using Jamovi Statistics for MacOS (Version 2.3.19). An ANCOVA) was conducted to compare changes in BDI-II scores from baseline to 30 days post-infusion across four treatment conditions: single ketamine infusion with psychotherapy, single ketamine infusion without psychotherapy, repeated ketamine infusions with psychotherapy, and repeated ketamine infusions without psychotherapy. The dependent variable was post-test BDI-II scores, with two independent variables (number of ketamine infusions and psychotherapy) and pre-test BDI-II scores as the covariate.

## 5. Conclusions

The findings from this study highlight the promise of ketamine infusion therapy as a rapidly acting intervention that expands the treatment landscape for individuals with TRD, a clinical population for whom therapeutic options remain limited and often ineffective. Unlike traditional antidepressants that may require several weeks to yield therapeutic benefits, ketamine has demonstrated the ability to produce significant reductions in depressive symptoms within hours to days, offering a critical window of relief for individuals experiencing severe or intractable depression. This presentation underscores the importance of advancing rigorous, multifaceted research into ketamine therapy. As a novel treatment modality, ketamine holds the potential to refine care for TRD; however, key questions remain regarding optimal dosing strategies, long-term efficacy, and relapse prevention. Addressing these questions will be vital to developing personalized and sustainable treatment protocols that maximize therapeutic benefit while minimizing risk.

While the findings are promising, the small sample size (*N* = 24) distributed across four groups significantly limits the statistical power of the analyses and warrants cautious interpretation, particularly regarding the effects attributed to psychotherapy. Although the study’s design reflects real-world clinical practice and offers valuable early insights into emerging treatments for TRD, the conclusions must be tempered by these methodological constraints.

Both treatment modalities appear to engage overlapping molecular and neurobiological mechanisms, particularly those involved in neuroplasticity, stress-response modulation, and inflammatory processes. While ketamine likely rapidly enhances synaptic connectivity and increases neurobiological receptivity, psychotherapy may facilitate long-term cognitive and emotional restructuring. Understanding how these biological effects converge may support a more cohesive and comprehensive model of treatment that optimizes outcomes and aligns with pharmacological and psychotherapeutic approaches. By continuing to explore ketamine’s molecular mechanisms, clinical applications, and integration with therapy, we can move toward more effective and individualized care for those suffering from TRD.

## Figures and Tables

**Table 1:** Therapeutic parameters of ketamine in clinical use.

Parameter	Details
Administration Routes	- Intravenous (IV): Most extensively studied; provides rapid onset (within minutes) and high bioavailability, allowing precise dose control [13,25]. - Intramuscular (IM): Used in outpatient or emergency settings; slower absorption than IV but still effective with fewer technical demands [26]. - Intranasal (IN): Esketamine formulation approved by FDA for TRD; convenient, non-invasive, but with variable bioavailability (~45–50%) and slower onset than IV [14,27]. - Oral/Sublingual: Limited by low and variable bioavailability (~20–25%) due to first-pass metabolism; used off-label with longer onset and duration [28].
Dosage Ranges	- IV subanesthetic doses: Typical dosing ranges 0.1–1.0 mg/kg; commonly 0.5 mg/kg infused over 40 min, producing rapid antidepressant effects without full anesthesia effects [13,27,29]. - IM: Typical dosing ranges 0.5–1.0 mg/kg, often used where IV access is not feasible [26]. - IN (esketamine): Dosing typically between 28 and 84 mg per administration; dosage tailored by severity and patient response [14,27]. - Oral: Approximate doses ~1–2 mg/kg, recognizing metabolism reduces bioavailability and potency [28].
Treatment Protocols	- Single IV administration: Demonstrates rapid onset of antidepressant and anti-suicidal effects, typically lasting 3–7 days, useful for acute crisis management [30,31]. - Repeated IV administration: Common regimens include 2–3 times per week for 2–4 weeks to sustain clinical benefit; repeated dosing shown to improve response durability and reduce relapse risk [14,31]. - Maintenance IV: Weekly or biweekly dosing for sustained remission; especially with intranasal esketamine, maintenance reduces relapse [14,26].
Targeted Clinical Conditions	- TRD: Ketamine has robust efficacy in patients unresponsive to conventional antidepressants, with rapid symptom reduction [13,26,29]. - MDD with suicidal ideation: Ketamine significantly reduces suicidal thoughts rapidly, making it valuable in emergency psychiatry [30]. - Post-traumatic stress disorder (PTSD): Emerging evidence supports benefits in PTSD symptom reduction [30,32]. - Obsessive–compulsive disorder (OCD): Early clinical trials suggest ketamine may reduce symptom severity temporarily [33]. - Bipolar depression: Demonstrates efficacy in depressive episodes, often adjunctive to mood stabilizers [9]. - Chronic pain syndromes: Off-label use for neuropathic and refractory pain; mechanisms likely linked to NMDA antagonism and central sensitization reduction [34].

**Table 2:** (**a**) Changes in BDI-II scores of patients in single infusion with psychotherapy (*n* = 9); (**b**) changes in BDI-II scores of patients in repeated infusions with psychotherapy (*n* = 5); (**c**) changes in BDI-II scores of patients in single infusion with no psychotherapy (*n* = 6); (**d**) changes in BDI-II scores of patients in repeated infusions with no psychotherapy (*n* = 4).

(**a**)	
Diagnosis pre-treatment	Frequency (Percentage)
Mild depression	3/9 (33%)
Moderate depression	5/9 (55%)
Severe depression	1/9 (11%)
Diagnosis post-treatment	Frequency (Percentage)
Clinically ‘normal’	5/9 (55%)
Mild depression	4/9 (44%)
Moderate depression	0/9 (0%)
Severe depression	0/9 (0%)
(**b**)	
Diagnosis pre-treatment	Frequency (Percentage)
Mild depression	0/5 (0%)
Moderate depression	0/5 (0%)
Severe depression	5/5 (100%)
Diagnosis post-treatment	Frequency (Percentage)
Clinically ‘normal’	0/5 (0%)
Mild depression	0/5 (0%)
Moderate depression	3/5 (60%)
Severe depression	2/5 (40%)
(**c**)	
Diagnosis pre-treatment	Frequency (Percentage)
Mild depression	2/6 (33%)
Moderate depression	2/6 (33%)
Severe depression	2/6 (33%)
Diagnosis post-treatment	Frequency (Percentage)
Clinically ‘normal’	2/6 (33%)
Mild depression	1/6 (16%)
Moderate depression	2/6 (33%)
Severe depression	1/6 (16%)
(**d**)	
Diagnosis pre-treatment	Frequency (Percentage)
Mild depression	0/4 (0%)
Moderate depression	2/4 (50%)
Severe depression	2/4 (50%)
Diagnosis post-treatment	Frequency (Percentage)
Clinically ‘normal’	0/4 (0%)
Mild depression	2/4 (50%)
Moderate depression	0/4 (0%)
Severe depression	2/4 (50%)

## Data Availability

The data presented in this study are available upon request from the corresponding author.

## Abbreviations

The following abbreviations are used in this manuscript:

TRD	Treatment-resistant depression
MDD	Major depressive disorder
SSRIs	Selective serotonin reuptake inhibitors
SNRIs	Serotonin–norepinephrine reuptake inhibitors
MAOIs	Monoamine oxidase inhibitors
TCAs	Tricyclic antidepressants
IV	Intravenous
IM	Intramuscular
NMDA	N-methyl-D-aspartate
AMPA	α-amino-3-hydroxy-5-methyl-4-isoxazolepropionic acid
BDNF	Brain-derived neurotrophic factor
mTOR	Rapamycin
CBT	Cognitive Behavioral Therapy
HPA	Hypothalamic–pituitary–adrenal
IL-6	Interleukin-6 (IL-6)
TNF-α	Tumor necrosis factor-alpha
PTSD	Post-traumatic stress disorder
KAP	Ketamine-assisted psychotherapy
KIC	Ketamine-induced cystitis
GAG	Glycosaminoglycan
ANCOVA	Analysis of covariance
